# Supplementary material for: Determinants of birth registration in India: Evidence from NFHS 2015–16
Source: PLoS One. 2021 Sep 2;16(9):e0257014. doi: 10.1371/journal.pone.0257014 (PMC8412296; doi:10.1371/journal.pone.0257014)
Supplement: Attachment — Submitted filename: Determinants of birth registration in India.docx [file pone.0257014.s002.docx]

Determinants of birth registration in India

1. P values of 0.000 should be written as p < 0.001 just like appeared in notes of the results
2. “insignificant” should read “not significant” (lines 276 and 280)
